# Supplementary material for: Cytotoxicity of Quantum Dots in Receptor-Mediated Endocytic and Pinocytic Pathways in Yeast
Source: Int J Mol Sci. 2024 Apr 26;25(9):4714. doi: 10.3390/ijms25094714 (PMC11083673; doi:10.3390/ijms25094714)
Supplement: Supplementary file 1 [file ijms-25-04714-s001.zip › ijms-2932541-supplementary.pdf]

Supplementary Data

Growth Assay

Comprehensive evaluation of the QDs' effects on yeast proliferation was performed by measuring the optical density (OD) of the yeast cultures, as illustrated in Supplementary Figure S1. A notable decline in yeast growth was observed after 6 hours following QD treatment, even at the minimal concentration of 4 μg/mL.

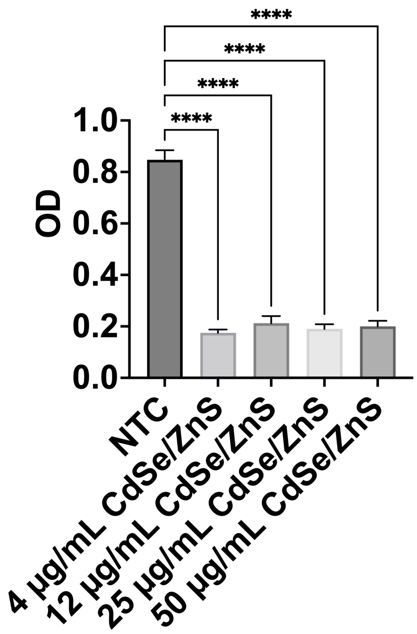

Supplementary Figure S1. Growth assay of yeast cells in varying concentrations of QDs (4 μg/mL, 12 μg/mL, 25 μg/mL, and 50 μg/mL). NTC - non-treated cells.

Assessment of Positive Control that interfere with the Endocytic pathway.

Based on our results of the lifespan of Cap1-GFP at the endocytic site (Figure 3), we conducted a positive control experiment using a *VPS1* mutant cells expressing Cap1-GFP. Time-lapse fluorescent videos were utilized to determine the lifespan of Cap1-GFP in the mutant strain as indicated in the method section.

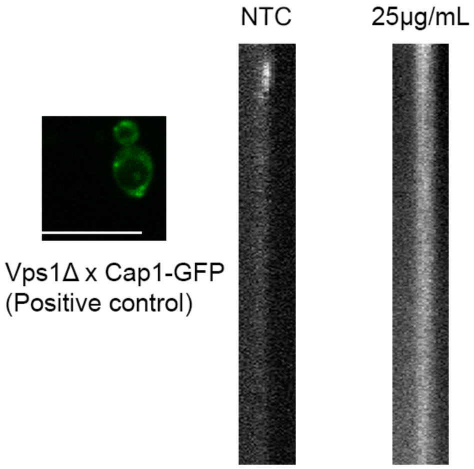

Supplementary Figure S2. (A) Representative image from Cap1-GFP in *VPS1*Δ in the presence of CdSe/ZnS QDs. The size bar is equivalent to 10 µm. (B) Kymograph of endocytic patch carrying Cap1-GFP without the presence of CdSe/ZnS QDs. (C) Kymograph of endocytic patch carrying Cap1-GFP in the presence of CdSe/ZnS QDs.

Characterization of CdSe/ZnS QDs Using Fluorometer Emission, and Dynamic Light Scattering (DLS).

Emission spectra of QDs upon 280 nm UV excitation.

The Fluorometer Emission technique was used to characterize the peak fluorescence of CdSe/ZnS QDs dispersed in an aqueous solution. QD samples of (5 mg/mL) of QDs were diluted to 5% (v/v) with 18 MΩ deionized water. A total of 180 µL of the diluted sample was then pipetted into a quartz cuvette, and the emission spectra were measured with a PTI Quantamaster spectrofluorometer with an excitation wavelength of 280 nm.

Our study confirms that emission spectrum of CZW-R-5 (red CdSe/ZnS QDs) in Supplementary Figure S3 displayed monodisperse peak with the emission peaks between 610 - 620 nm, while CZW-G-5 (green CdSe/ZnS QDs) has a monodisperse peak with hydrodynamic diameter around 540-560 nm (Supplementary Figure S3). These findings align with manufacturer specifications.

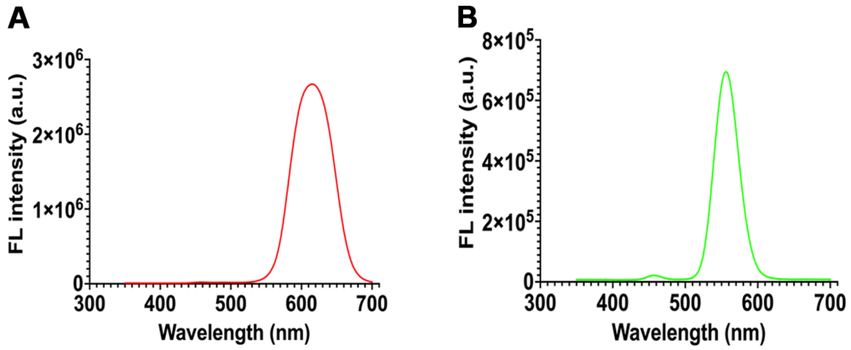

Supplementary Figure S3 (A) Emission spectrum of red CdSe/ZnS QDs (CZW-R-5) upon 280 nm UV excitation. (B) Emission spectrum of green CdSe/ZnS QDs (CZW-G-5) upon 280 nm UV excitation.

Dynamic Light Scattering

The dynamic light scattering (DLS) technique was used to characterize the hydrodynamic size of red and green CdSe/ZnS QDs dispersion in an aqueous solution. The stock concentrations (5 mg/mL) of QDs were diluted to 5% (v/v) with 18 MΩ deionized water. A total of 180 µL of the diluted sample was then pipetted into a quartz cuvette and the hydrodynamic particle size was measured with a Zetasizer Ultra (Malvern Panalytical Ltd., Malvern, UK).

The DLS results of CZW-R-5 (red CdSe/ZnS QDs) in Supplementary Figure S4 has a monodisperse peak with hydrodynamic diameter between 12-13 nm (Supplementary Figure S4), while CZW-G-5 (green CdSe/ZnS QDs) displayed monodisperse peak at hydrodynamic diameters between 13-14 nm, (Supplementary Figure S4).

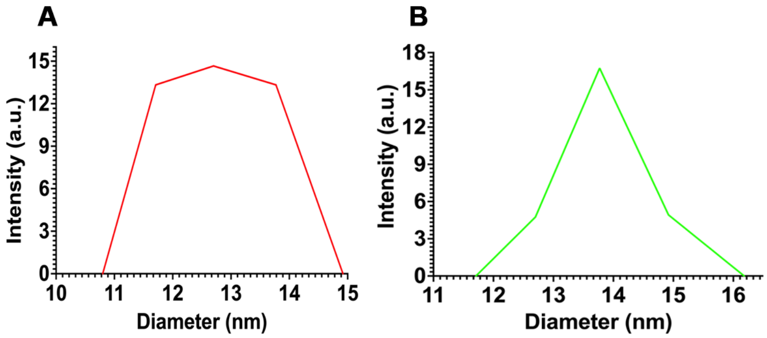

Supplementary Figure S4 (A) Intensity size distribution of CZW-R-5 (red CdSe/ZnS QDs) obtained by Dynamic Light Scattering. (B) Intensity size distribution of CZW-G-5 (green CdSe/ZnS QDs) obtained by Dynamic Light Scattering.
